# Analysis of the transcriptomic features of microsatellite instability subtype colon cancer

**DOI:** 10.1186/s12885-019-5802-2

**Published:** 2019-06-20

**Authors:** Haiwei Wang, Xinrui Wang, Liangpu Xu, Ji Zhang, Hua Cao

**Affiliations:** 1 Fujian Key Laboratory for Prenatal Diagnosis and Birth Defect, Fujian Provincial Maternity and Children’s Hospital, affiliated hospital of Fujian Medical University, FuZhou, FuJian China; 2 Key Laboratory of Technical Evaluation of Fertility Regulation for Non-human Primate, National Health and Family Planning Commission, FuZhou, FuJian China; 3 State Key Laboratory for Medical Genomics, Shanghai Institute of Hematology, Rui-Jin Hospital Affiliated to School of Medicine, Shanghai Jiao Tong University, Shanghai, China

**Keywords:** PD-1, IFN-γ, MSI, Wnt-β-catenin signaling pathway, TGFβ signaling pathway, M1 macrophage cells

## Abstract

**Background:**

Programmed cell death protein 1(PD-1) blocking antibodies have been used to enhance immunity in solid tumors and achieve durable clinical responses with an acceptable safety profile in multiple types of cancer. However, only a subset of patients could benefit from PD-1 blockade therapy. Prognostic information including PD-1 ligand (PD-L1) expression, IFN-γ expression signature, tumor mutational burden, and microsatellite instability (MSI) have been evaluated for patients who are selected to receive immune checkpoint therapeutic treatment. Yet the relationship of those biomarkers in determining immune checkpoint therapy is largely unknown.

**Methods:**

Immune-profiles of MSI subtype colon cancer were identified from integrating published MSI associated gene expression data. The enriched pathways and transcription factors were analyzed by GSEA assay. The infiltrations of immune cell types into MSI subtype colon cancer tissues were determined by CIBESORT assay.

**Results:**

In the MSI subtype colon cancer patients, PD-L1, IFN-γ and IFN-γ associated genes are highly expressed. And all those genes are favorable effects in colon cancer progress. In addition, we find that Wnt-β-catenin and TGFβ signaling pathways which are two important factors inhibiting PD-1 checkpoint blockade therapy are negatively related with MSI status. We also identify that the immune-profiles in MSI subtype colon cancer are contributed by M1 macrophage infiltration in the tumor environment.

**Conclusions:**

Our results provide the detailed underlying mechanisms of MSI subtype cancer patients are sensitive to PD-1 checkpoint blockade.

**Electronic supplementary material:**

The online version of this article (10.1186/s12885-019-5802-2) contains supplementary material, which is available to authorized users.

## Background

The MisMatch Repair (MMR) repair system copes with nucleotide mutations, insertions and deletions occurred during DNA replication, including MLH1, MSH2, MSH6 and PMS2 genes [1]. Germ line mutations in MMR genes are associated with Lynch syndrome, which have high probability to develop colon cancer. Somatic mutations of MMR genes or loss of MMR genes by hyper-methylation in patients is associated with MSI status [2]. Clinical results across 12 different tumor types have shown that MMR deficient patients are more sensitive to immune checkpoint blockade therapy [3, 4]. And FDA has approved MSI as a biomarker to ensure the success of cancer immunotherapy in multiple types of cancer. Although, the gene expression signature of MSI subtype colon cancer patients was previously studied [5–7], most of those studies are not focusing on how those changed genes influence the cancer response to checkpoint blockade immunotherapy.

PD-L1, IFN-γ expression and IFN-γ associated immune gene signature are critical factors in determining the sensitivity of anti-PD-1 therapies [8, 9]. Non-responders to PD-1 checkpoint blockade immunotherapy are associated with defects in the pathways involved interferon receptor signaling pathway [10–12]. It has been reported that MSI subtype of colorectal cancer exhibits an active expression of checkpoint molecules, like PD-L1 and IFN-γ in the tumor microenvironment [13]. However, the immune-profiles in MSI subtype colon cancer need to be further studied in a globe expression manner.

T cells distribution is a key factor that influences the response to cancer immunotherapy [14]. Immunotherapy responding patients are characterized by the presence of CD8 T cells in the tumor environment. Conversely, patients who do not respond to anti-PD-1 antibodies present devoid of T cells. This default in CD8 T cell infiltration in tumor has been observed in lung, pancreatic, and ovarian carcinomas [15]. In the PD-1 checkpoint blockade responding tumor microenvironment, activated T cells, as well as natural killer cells produce IFN-γ. IFN-γ directly up regulates PD-L1 expression [16]. Macrophage cells play important roles in CD8 T cells tumor surveillance and tumors response to anti-PD-1 treatment [17]. However, the roles of macrophage infiltrations in the tumor environment in MSI subtype colon cancer are largely unknown.

TGFβ [18, 19] and Wnt-β-catenin [20, 21] are new identified signaling pathways associated with PD-1 checkpoint blockade therapy. Inhibition of TGFβ or Wnt-β-catenin signaling increases the benefits of immune therapies on tumor patients. However, the relationship between MSI status and TGFβ/Wnt-β-catenin signaling pathway have not been studied.

Here, we attempt to address the relationship between MSI status and immunotherapy sensitivity by integrating published MSI associated expression datasets in colon cancer patients. We find that PD-L1 mRNA is highly expressed in the MSI subtype colon cancer patients. And MSI expression signature contains high IFN-γ expression and IFN-γ associated immune gene activation. While the TGFβ and Wnt-β-catenin signaling pathways are inactivated in MSI subtype colon cancer patients. At last, we find that the immune-profiles in MSI subtype colon cancer are contributed by M1 macrophage infiltration in the tumor environment.

## Methods

### Data collection

Gene expression series matrix of colon cancer samples with clinical annotation MSI or MSS status were downloaded from GEO website (https://www.ncbi.nlm.nih.gov/geo/) with GEO number GSE13067, GSE13294, GSE18088, GSE24551, GSE26682, GSE39084 and GSE41258. Brief descriptions of the 7 datasets could be found in Additional file 1: Figure S1a.

Gene expression series matrix of 6 cohorts of colon cancer patients used for Immune Cellular Fraction Estimation were downloaded from GEO website with GEO number GSE14333, GSE17536, GSE24551, GSE33113, GSE39084 and GSE39582. Brief descriptions of the 6 datasets could be found in Additional file 1: Figure S1c.

Colon cancer samples with gene expression and survival data were available at GEO website with GEO number GSE39582 and GSE24551.

### GEO data processing

All the expression profiles were processed separately. A probe was removed if it was not corresponded gene symbol, and the expression values were averaged if multiple probes corresponded to the same gene symbol using R software “plyr” package.

### Gene set enrichment analysis (GSEA)

Gene set enrichment analysis was performed using GSEA 2.0 software. CP: KEGG gene sets (CP: KEGG) and TF transcription factor targets (TFT) gene sets were downloaded from the GSEA Web site (http://www.broad.mit.edu/gsea/index.html). Genes ranked by signal-to-noise ratio, and statistical significance was determined by 1000 gene set permutations.

### Gene ontology (GO) enrichment analysis

Gene Ontology (GO) enrichment analysis was performed using DAVID website (https://david.ncifcrf.gov).

### Survival analysis

Kaplan-Meier estimator was applied to identify the influence of gene expression on overall survival or relapse free survival using “survival” package in the R statistics software.

### The consensus molecular subtypes (CMS) of colorectal cancer classification

Colon cancer patients from GSE13924, GSE18088 and GSE39084 datasets were divided into CMS1, CMS2, CMS3 and CMS4 subtypes by R software “CMScaller” package.

### Heatmap presentation

Heatmaps were created by R software “pheatmap” package.

### Drug sensitivity analysis

Human colon cancer cell lines with PARP inhibitors sensitivity and expression data were downloaded from Genomics of Drug Sensitivity in Cancer (http://www.cancerrxgene.org/). The colon cancer cell lines were divided into MSI or MSS two group based on the cell lines annotation.

### Immune cellular fraction estimates

The relative fractions of 22 immune cell types within the leukocyte compartment were estimated using “CIBERSORT” package in the R statistics software [22]. Statistical significance was determined by 10 gene set permutations.

### Spearman correlation

Spearman correlation was used to study the correlation between PD-L1 expression and 22 immune cell types cibersort fraction or MLH1 expression and 22 immune cell types cibersort fraction.

### Statistical analysis

The box plots and contingency graphs were generated from prims5.0. Statistical analysis was performed using the Student’s t test or Chi-square test. *P* value less than 0.05 was chosen to be statistically significant difference unless specifically notified.

## Results

### PD-L1 and IFN-γ signature genes are activated in MSI subtype colon cancer patients

To address the mechanistic basis of MSI status in determining immune checkpoint therapy sensitivity, we analyzed colon cancer patients with expression data and MSI annotation from previously published GEO datasets. Totally, 830 patients were collected from 7 published datasets based on 3 platforms. Two hundred seven patients are MSI or MSI high, 623 patients are microsatellite stability (MSS) (Additional file 1: Figure S1a). MSI patients are characterized by epigenetic silencing of MLH1 or mutation in one of the MMR genes MLH1, MSH2, MSH6 or PMS2. To validate the MSI status annotation in the datasets, we analyzed the MLH1, MSH2, MSH6 and PMS2 expression in those patients. In all the 7 datasets, MLH1 mRNA was significantly down regulated in MSI patients than MSS patients (Additional file 1: Figure S1b). However, MSH2, MSH6 and PMS2 mRNA was not significantly down regulated in MSI patients.

**Fig. 1:**
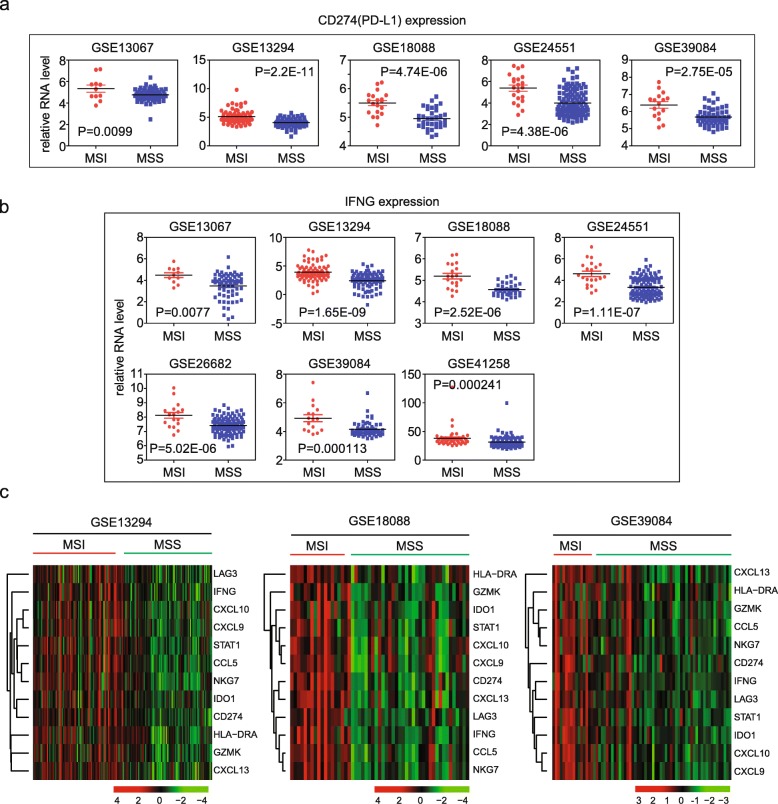
PD-L1 and IFN-γ signature genes are activated in MSI subtype colon cancer patients. **a** Box plots showed the PD-L1 (CD274) expression in 5 GEO datasets. *P* values showed the difference of PD-L1expressions between MSI and MSS colon cancer patients determined by Student’s t test. **b** Box plots showed the IFN-γ (IFNG) expression in 7 GEO datasets. **c** Un-supervised clustering heatmaps showed the IFN-γ signature genes in MSI and MSS colon cancer patients in GSE13294, GSE18088 and GSE39084 datasets

First, we wanted to test PD-L1 expression in the colon cancer patients. Among five datasets GSE13067, GSE13294, GSE18088, GSE24551 and GSE39084, PD-L1 (CD274 for PD-L1 gene symbol) was highly expressed in MSI patients than MSS patients (Fig. 1a). No PD-L1 probe was designed in GSE26682 and GSE41258 datasets. Those observations provided the clue that MSI positive patients were sensitive to PD-1 blockage was due to the high mRNA expression of PD-L1 in MSI subtype colon cancer.

IFN-γ and IFN-γ associated immune gene signatures are critical factors in determining the response of anti-PD-1 therapies [9]. Defects in IFN-γ pathway genes induce acquired resistance to PD-1 blockade immunotherapy [23, 24]. Results showed that IFN-γ was highly expressed in MSI patients in all 7 datasets (Fig. 1b). Except IFN-γ itself, IFN-γ associated immune gene signature including IDO1, CXCL10, CXCL9, HLA-DRA and STAT1 and IFN-γ expanded immune gene signature LAG3, CCL5, NKG7, GZMK and CXCL13 were also increased in MSI patients as presented through gene expression heatmaps in GSE13924, GSE18088 and GSE39084 datasets (Fig. 1c).

### Immune associated signaling pathways are activated in MSI subtype colon cancer patients

**Fig. 2:**
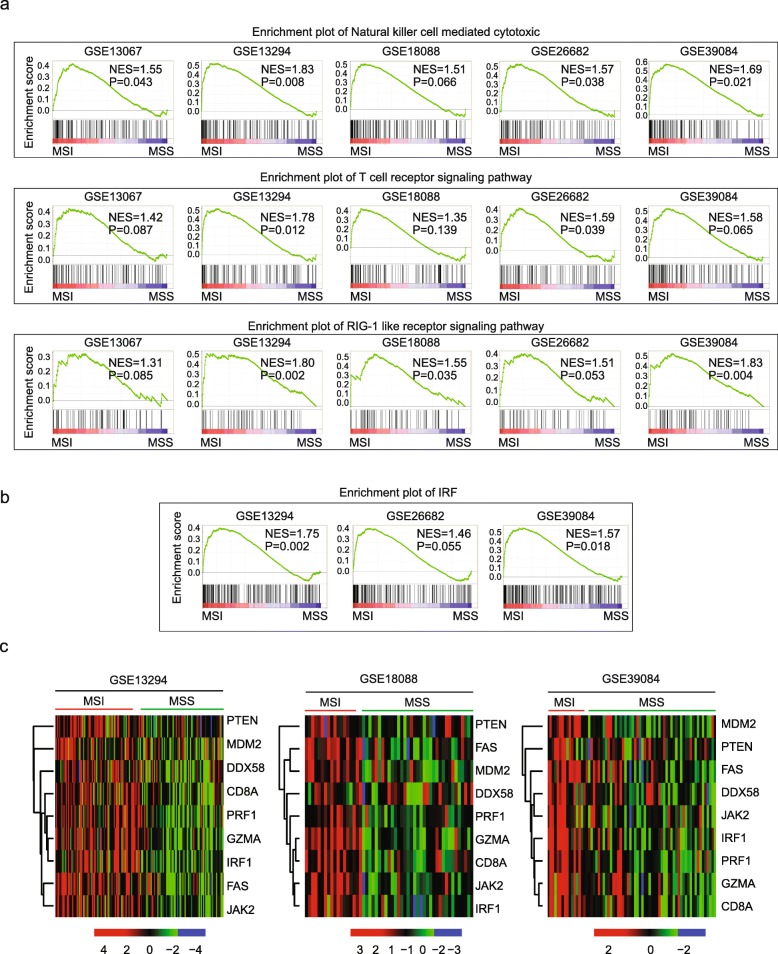
Immune associated signaling pathways and transcription factor IRF1 are activated in MSI subtype colon cancer patients. **a** Enrichment plots showed the natural killer cell mediated cytotoxic pathway, T cell receptor signaling pathway and RIG-1 like receptor signaling pathway in 5 datasets. Enrichment of NES and *P* values were shown. **b** Enrichment plots showed the transcription factor IRF in 3 datasets. **c** Un-supervised clustering heatmaps showed the expressions of IRF1 and genes from immune associated signaling pathways in MSI and MSS colon cancer patients in GSE13294, GSE18088 and GSE39084 datasets

To further reveal the transcription property of MSI status, we identified the signaling pathways enriched in MSI subtype colon cancer patients using GSEA assay. Among all the enriched signaling pathways, natural killer cell mediated cytotoxic, T cell receptor signaling pathway and RIG-1 like receptor signaling pathway were highly enriched in at least 2 datasets (Fig. 2a). Particularly, natural killer cell mediated cytotoxic signaling pathway was most significantly enriched in 5 datasets.

Natural killer cell mediated cytotoxic signaling pathway presents an important executor mediating PD-1 blockage to eliminate tumor cells. The significant activation of natural killer cell mediated cytotoxic pathway suggested that MSI tumor cells may activate a more dramatic immunity function to eliminate tumor cells upon immunotherapy treatment. T cell receptor signaling pathway and RIG-1like receptor signaling pathway are important regulators of IFN-γ production and antiviral gene expression [25]. The activated signaling pathways were greatly consistent with the high expression of IFN-γ signature genes in MSI subtype colon cancer patients.

Next, we showed that T cell receptor signaling pathway and RIG-1like receptor signaling pathway associated genes granzyme A (GZMA), perforin (PRF1), T cell receptor CD8A and DDX58 (RIG-1) were increased in MSI subtype colon cancer patients as presented through gene expression heatmaps in GSE13924, GSE18088 and GSE39084 datasets (Fig. 2c). We also noticed that TP53 signaling pathway and TP53 target genes PTEN, FAS and MDM2 were also activated in MSI patients (Fig. 2c and Additional file 1: Figure S2). Loss of PTEN expression in tumor cells promotes resistance to immunotherapy in both melanoma and uterine leiomyosarcoma by decreasing T cell infiltration [26, 27]. Based on those results, we speculated that the MSI subtype colon cancer patients were more easily induced into an immune activated status and were more sensitive to PD-1 blocking antibodies induced enhanced immunity to eliminate solid tumor cells.

### Transcription factor IRF1 is activated in MSI subtype colon cancer patients

Except signaling pathways, the transcription factors enriched in MSI subtype colon cancer patients were also identified. No transcription factor was enriched in at least 3 datasets. The enriched transcription factors varied from dataset to dataset, suggesting a diverse property of MSI subtype colon cancer. However, we noticed that IRF (Interferon regulatory factor) was highly enriched in GSE13294 and GSE39084, and less significantly enriched in GSE26682, representing the most frequent enriched transcription factor (Fig. 2b). Results have suggested that IRF factors orchestrate immune responses and destruction of allogeneic organ transplants [28]. IRF contains nine families, from IRF1 to IRF9. In the MSI subtype colon cancer patients’ datasets, we showed that IRF1 was highly up regulated in MSI patients (Fig. 2c). Interferon-receptor–associated Janus kinase 2 (JAK2) was also highly expressed in MSI subtype colon cancer patients (Fig. 2c), suggesting the activation of IFN-γ-JAK2-IRF1 axis.

### Wnt-β-catenin and TGFβ signaling pathways are inactivated in MSI subtype colon cancer patients

**Fig. 3:**
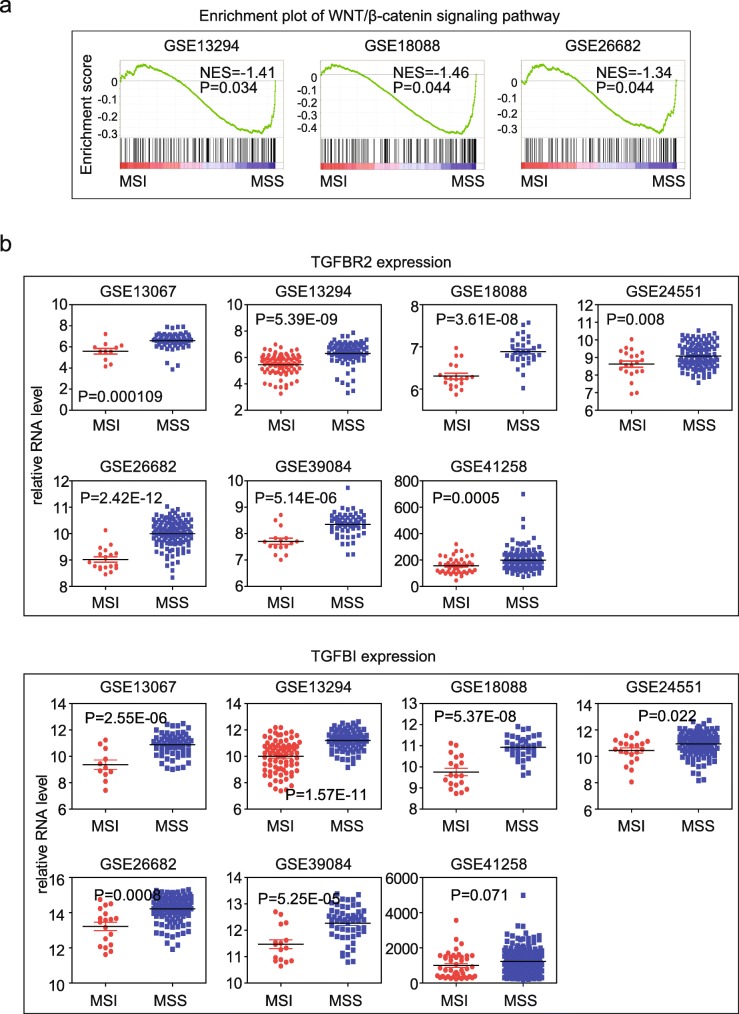
Wnt-β-catenin and TGFβ signaling pathways are inactivated in MSI subtype colon cancer patients. **a** Enrichment plots of the Wnt-β-catenin signaling pathway in 4 datasets. **b** Box plots showed the TGFβ associated TGFBR2 and TGFB1 expressions in 7 GEO datasets. *P* values showed the difference of genes expression between MSI and MSS colon cancer patients and were determined by Student’s t test

Contrast to the IFN-γ signature, which promotes immunotherapeutic efficiency, Wnt-β-catenin [20, 21] and TGFβ [18, 19] signaling pathways are two important factors inhibiting PD-1 checkpoint blockade therapy. Inhibition of Wnt-β-catenin and TGFβ signaling could increase the benefit of immune therapies on tumor patients. We noticed that Wnt-β-catenin signaling was significantly negatively related to MSI status in GSE13294, GSE18088 and GSE26682 datasets, implying the inhibitory roles of Wnt-β-catenin signaling pathway (Fig. 3a).

We did not observe significantly enriched TGFβ signaling pathway in any of the 7 datasets. But we found that TGFβ signaling associated genes TGFBR2 and TGFBI was highly expressed in MSS subtype of colon cancer (Fig. 3b), suggesting the potential inhibition of TGFβ signaling pathway in MSI subtype colon cancer patients.

### IFN-γ signature genes are associated with favorable outcomes in colon cancer

**Fig. 4:**
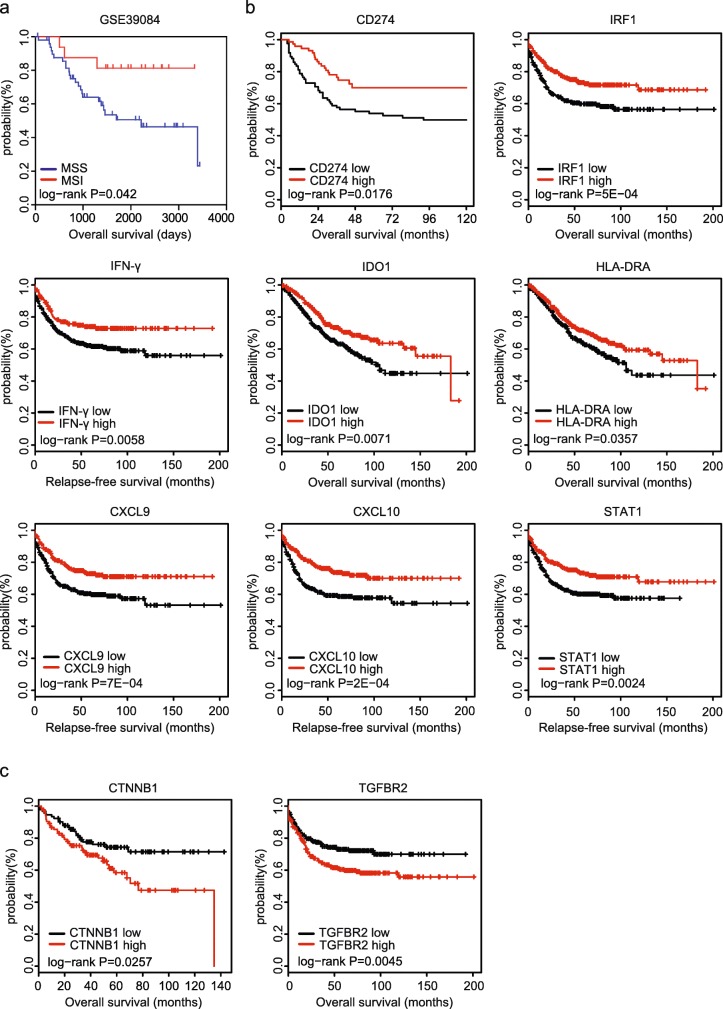
IFN-γ signature genes are associated with favorable outcomes in colon cancer. **a** Relationships of the MSI/MSS status and overall survival were analyzed from GSE39084. Kaplan Meier survival analysis was used to compare between MSI colon cancer patients and MSS colon cancer patients. Log-rank test estimated *p* value. **b** Relationships of the PD-L1, IRF1, IFN-γ and IFN-γ signature genes IDO1, HLA-DRA, CXCL9, CXCL10, STAT1 expressions and colon cancer relapse free or overall survival were analyzed from GSE39582 and GSE24551 datasets. Kaplan Meier survival analysis was used to compare colon cancer patients with high gene expression to patients with low genes expression. Log-rank test estimated *p* value. **c** Relationships of β-catenin (CTNNB1), TGFBR2 expression and colon cancer overall survival were analyzed

Clinically, MSI colon cancer patients have favorable prognosis than MSS colon cancer patients [2]. Indeed, MSS subtype colon cancer patients in GSE39084 were with low overall survival rate (Fig. 4a). Since, PD-L1, IRF1, IFN-γ and IFN-γ associated immune gene were important features of MSI status, we then investigated the roles of those genes in colon cancer progress. We collected colon cancer patient expression profiles containing prognostic information from public database GSE39582 and GSE24551. The kaplan-meier survival analysis was used to show the different prognosis between high gene expression group and low gene expression group of colon cancer patients. PD-L1, IRF1, IFN-γ and IFN-γ associated immune signature genes IDO1, CXCL10, CXCL9, HLA-DRA and STAT1 all had similar prognostic effects. High expressions of those genes were associated with favorable prognosis (Fig. 4b). However, in colon cancer patients, β-catenin (CTNNB1) and TGFBR2 were all unfavorable effects on tumor progress. Patients with high expression of CTNNB1 or TGFBR2 were with low overall survival rate (Fig. 4c). Those results consisted with the inhibitory roles of Wnt-β-catenin signaling and TGFβ signaling in MSI subtype colon cancer patients.

### PD-L1 and IFN-γ are activated in CMS1 colon cancer subtype patients

**Fig. 5:**
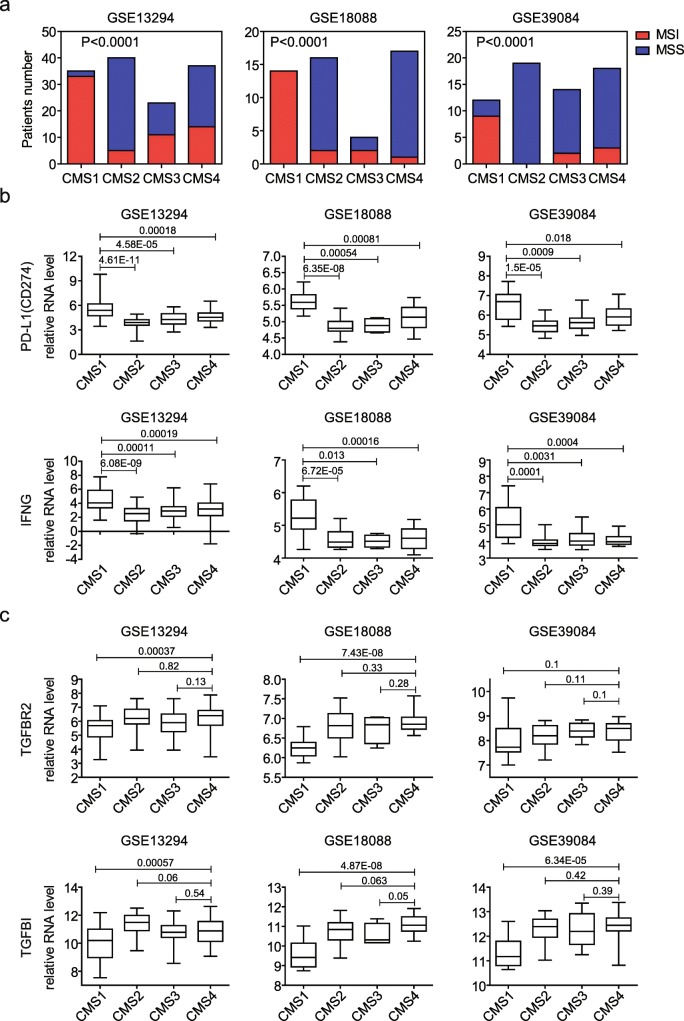
PD-L1 and IFN-γ are activated in CMS1 colon cancer subtype patients. **a** Contingency graphs showed the number of MSI or MSS subtype colon cancer patients in each CMS subgroup. *P* values showed the difference between MSI (red) and MSS (blue) colon cancer patients determined by Chi-square test. **b** Box plots showed the PD-L1 and IFN-γ expressions in different CMS subgroups in GSE13294, GSE18088 and GSE39084 datasets. **b** Box plots showed the TGFβ associated TGFBR2 and TGFB1 expressions in in different CMS subgroups in GSE13294, GSE18088 and GSE39084 datasets

Previously, multiple colon subtypes had been identified with different methods based on the DNA methylation, DNA somatic copy number alterations (SCNA) or gene expression profiling [29–31]. However, the expressions of PD-L1 and IFN-γ in those different subtypes of colon cancer patients were not clear. We used the consensus molecular subtypes (CMS) of colorectal cancer classification to demonstrate the PD-L1 and IFN-γ expressions. Using CMScaller [32], patients from GSE13924, GSE18088 and GSE39084 datasets were divided into CMS1, CMS2, CMS3 and CMS4 subtypes. The majority of MSI colon patients were in CMS1 subtype. While, the majority of MSS colon patients were in CMS2 and CMS4 subtypes (Fig. 5a). Particularly, in GSE18088, CMS1 subtype was all MSI colon patients. And in GSE39084, CMS2 was all MSS colon patients (Fig. 5a). Those results were consistent with the previous observation that CMS1 encompassed the most of MSI colon cancer patients [30].

Next, we determined the PD-L1 and IFN-γ expression in CMS1, CMS2, CMS3 and CMS4 subtypes from the GSE13924, GSE18088 and GSE39084 datasets. Consistent with the high proportion of MSI patients in CMS1 subtype colon cancer, PD-L1 and IFN-γ were highly expressed in CMS1 than other three CMS subtypes (Fig. 5b). Those results further highlighted the immune infiltration and activation property of CMS1.

Previous results showed that CMS2 subtype colon cancer was characterized by WNT activation and CMS4 was characterized by the activation of TGFβ signaling pathway [30]. Since, the majority of MSS colon patients were in CMS2 and CMS4 subtypes, it was expected that Wnt-β-catenin and TGFβ signaling pathways were enhanced in MSS subtype colon cancer patients (Fig. 3a) and TGFβ signaling associated genes TGFBR2 and TGFBI was highly expressed in CMS4 subtypes of colon cancer (Fig. 5c), particularly compared with CMS1 subtype. Those results also confirmed the fact that TGFBR2 and TGFBI genes were highly expressed in MSS not because of the Hypoxia/hypoxia-inducible factor signaling pathway but because of the potential inhibition of TGFβ signaling pathway in MSI subtype colon cancer patients.

### IFN-γ signature genes are not activated in MSI subtype colon cancer cell lines

**Fig. 6:**
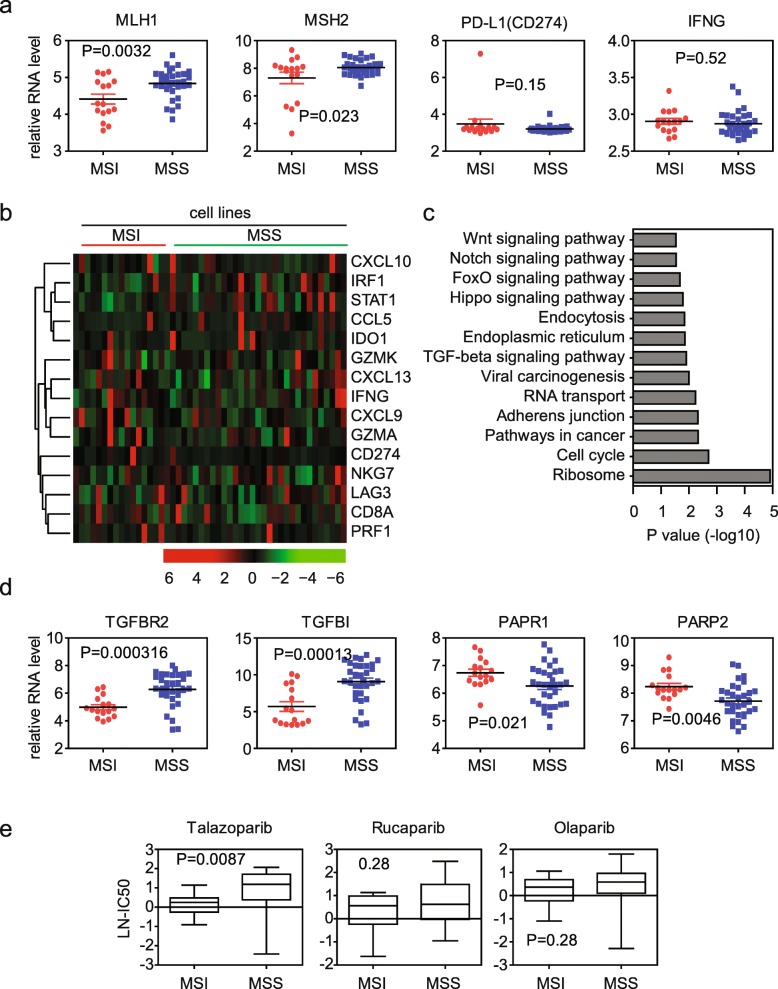
IFN-γ signature genes are not activated in MSI subtype colon cancer cell lines. **a** Box plots showed the MLH1, MSH2, PD-L1 (CD274) and IFNG expressions in colon cancer cell lines. *P* values showed the difference of gene expression between MSI and MSS colon cancer cell lines determined by Student’s t test. **b** Un-supervised clustering heatmaps showed IFN-γ signature genes expressions in MSI and MSS colon cancer cell lines. **c** Functional pathways enrichment analysis from the regulated genes in MSI colon cancer cell lines using DAVID. The most enriched pathways were demonstrated. **d** Box plots showed the TGFBR2, TGFB1, PARP1 and PARP2 expressions in colon cancer cell lines. **e** Box plots showed the LN-IC50 of PARP inhibitors talazoparib, rucaparib and olaparib in MSI or MSS colon cancer cell lines

Next, we tested the immune-profiles in MSI colon cancer cell lines. Colon cancer cell lines with gene expression data and MSI annotation were downloaded from the Genomics of Drug Sensitivity in Cancer [33]. Box plots showed the MMR genes MLH1 and MSH2 expression in colon cancer cell lines (Fig. 6a). MLH1 and MSH2 mRNA was significantly down regulated in MSI than MSS subtype colon cancer cell lines. However, unlike in MSI subtype colon cancer patients, we did not observe the high expression of PD-L1 and IFN-γ in MSI colon cancer cell lines (Fig. 5a). Un-supervised clustering of gene expression heatmaps showed that there were no differences of IFN-γ signature genes expression between MSI and MSS subtype colon cancer cell lines (Fig. 6b). Functional pathway enrichment analysis from the regulated genes in MSI subtype colon cancer cell lines showed that no significant immune associated pathways was enriched (Fig. 6c). Those results implied that the activated immune-profiles in MSI colon cancer patients were contributed by the immune tumor environment.

Globe different gene expression profiles between MSI and MSS in colon cancer cell lines was further identified (Additional file 1: Figure S2a). Overlapped with the colon cancer patient’s data, we found that some genes were both activated in MSI colon cancer patients’ tissue and MSI colon cancer cell lines. For instance, TGFβ signaling associated genes TGFBR2 and TGFBI were both not enhanced in MSI colon cancer patients’ tissue and MSI colon cancer cell lines (Fig. 3b and 6d). Importantly, we found that TGFβ and Wnt signaling pathway were significantly enriched from MSI colon cancer cell lines regulated genes (Fig. 6c). Those results implied that the inactivation of TGFβ and Wnt signaling pathways in MSI subtype colon cancer patients were due to the tumor cell itself.

### MSI subtype colon cancer cell lines are particular sensitive to PARP inhibitor talazoparib

PARP1 and PARP2 are the main repair system for the DNA single strand breaks damage [34]. PARP1 and PARP2 were both activated in MSI subtype colon cancer patients’ tissue and cell lines (Fig. 6d and Additional file 1: Figure S2b). Those results implied that MSI subtype colon cancer patients may be sensitive to PARP inhibitor treatment. To perform this analysis, we used the Genomics of Drug Sensitivity in Cancer database, which included response of colon cancer cells to PARP inhibitors treatment. We found that one PARP inhibitor talazoparib but not rucaparib and olaparib was particularly targeting on MSI subtype colon cancer cells (Fig. 6e).

### MSI subtype colon cancer patients are with high M1 macrophage infiltration

**Fig. 7:**
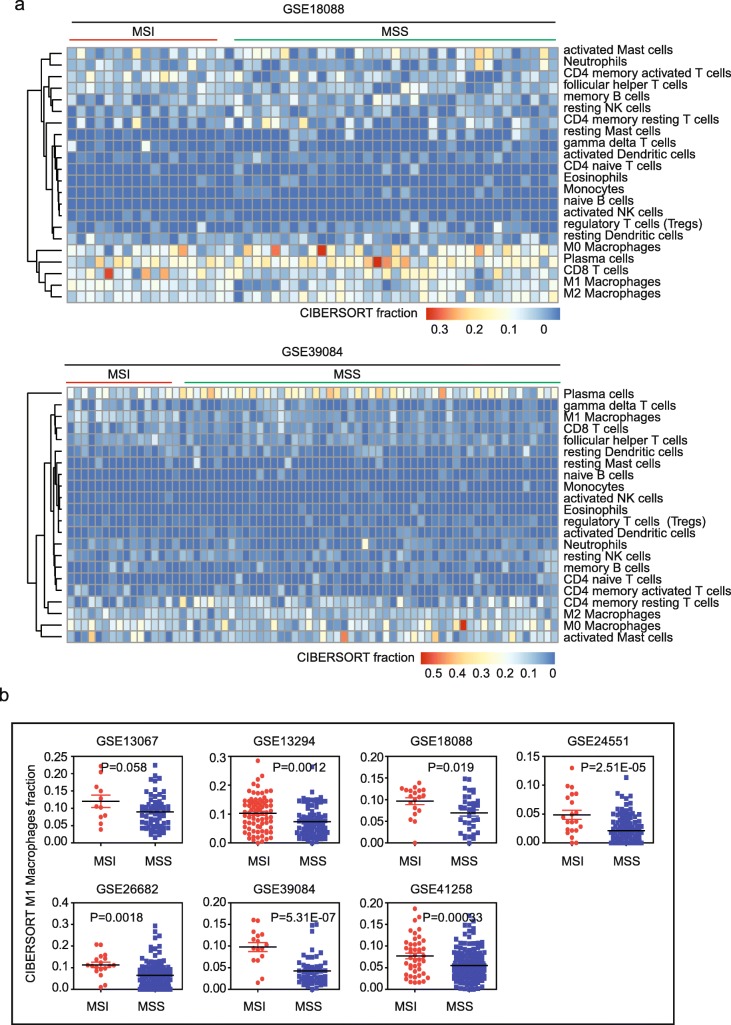
MSI subtype colon cancer patients are with high M1 macrophage infiltration. **a** Un-supervised clustering heatmaps showed the cibersort fraction of 22 immune associated cell types in MSI and MSS colon cancer patients in GSE18088 and GSE39084 datasets. **b** Box plots showed the cibersort fraction of M1 macrophage cells in 7 GEO datasets. *P* values showed the difference of cibersort fraction of M1 macrophage cells between MSI and MSS patients determined by Student’s t test

Recent studies suggested that the immune cell infiltration could increase PD-L1 expression in cancer cells and enhance the response to anti-PD-1 therapies [13]. To characterize which immune associated cell types in the tumor environment contributing the activated immune-profiles in MSI subtype colon cancer, we scored immune expression signatures to determine the fraction of immune associated cell types by Cibersort assay [22, 35]. Un-supervised clustering heatmaps showed the cibersort fraction of 22 immune associated cell types in MSI and MSS colon cancer patients in GSE18088, GSE39084 and GSE13294 (Fig. 7a and Additional file 1: Figure S3a). We found that in the colon tumor environment, the most infiltrated cell type was plasma cells. And M0 macrophage cells were also significantly infiltrated into the tumor environment. However, we did not find significant difference in cell infiltration of plasma cells or M0 macrophage cells between MSI and MSS colon cancer patients.

By studying the 22 immune associated cell types, we found that the cibersort fraction of M1 macrophages cell was greatly higher in MSI than MSS subtype colon cancer patients in all 7 GEO datasets (Fig. 7b). CD8 T cells (Additional file 1: Figure S3b) and γδ T cells (Additional file 1: Figure S3c) were also highly infiltrated into MSI colon tumor environment. Combined those results suggested that the highly activated immune-profiles in MSI subtype colon cancer were contributed by high infiltration of M1 macrophages cells, CD8 T cells and γδ T cells.

### M1 macrophages infiltration fraction is negatively associated with MLH1 expression but positively associated with PD-L1 expression in colon cancer patients

**Fig. 8:**
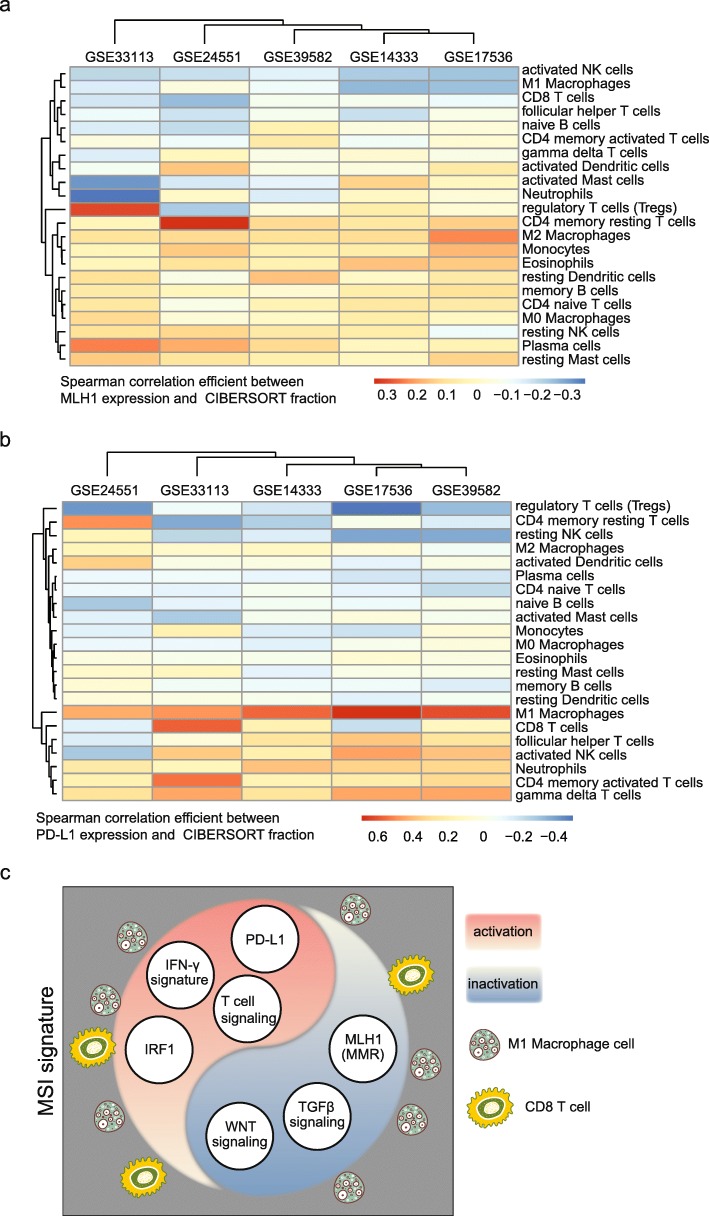
M1 macrophage infiltration fraction is negatively associated with MLH1 expression but positively associated with PD-L1 expression in colon cancer patients. **a** Un-supervised clustering heatmaps showed the spearman correlation efficient between MLH1 expression and cibersort fraction of 22 immune associated cell types from 6 colon cancer patients’ datasets. **b** Un-supervised clustering heatmaps showed the spearman correlation efficient between PD-L1 expression and cibersort fraction of 22 immune associated cell types from 6 colon cancer patients’ datasets. **c** A graphical picture showed the MSI signature with the inactivation of MMR system, TGFβ signaling pathway and Wnt signaling pathway, but with the activation of PD-L1 expression, IFN-γ signature, IRF1 transcription factor activation and T cell signaling activation. The high infiltrations of M1 macrophage and CD8 T cells may contribute those signatures in MSI subtype colon cancer patients

To further confirm the contributions of M1 macrophage cells to the activation of immune-profiles in MSI subtype colon cancer patients, we studied other 1338 colon cancer patients with gene expression profiles from 5 GEO datasets (Additional file 1: Figure S1c). We used MLH1 expression level as an estimator of MSI status. In each GEO dataset, we first determined the infiltrated fraction of 22 immune associated cell types using Cibersort. Then we determined the correlation between cell fraction and MLH1 expression. Un-supervised clustering heatmaps of the spearman correlation efficient between MLH1 expression and cibersort fraction of 22 immune associated cell types from 6 colon cancer patients’ datasets suggested that M1 macrophages infiltration fraction was highly negatively associated with MLH1 expression (Fig. 8a). Similar procedures were used to determine the correlation efficient between PD-L1 expression and cibersort fraction of 22 immune associated cell types. We found that PD-L1 expression was highly positively associated with M1 macrophages infiltration fraction in colon cancer patients (Fig. 8b).

Overall, in our data analysis, we identified multiple expression characteristics in the MSI subtype of colon cancer, for instance, inactivation of MMR system, TGFβ signaling pathway and Wnt-β-catenin signaling pathway, and the activation of PD-L1, IFN-γ signature, IRF1 transcription factor and T cell signaling pathway. The high infiltration of M1 macrophage and CD8 T cells may contribute those signatures in MSI subtype colon cancer patients (Fig. 8c).

## Discussion

Blocking the immune checkpoint pathways presents new generation of cancer therapy. Despite the durable clinical response and acceptable safety profile, only a subset of patients could benefit from immune blockade therapies. Identification of clinically responsive biomarkers is important to ensure the success of cancer immunotherapy. Multiple clinical studies have shown that patients with MSI are more sensitive to anti-PD-1 antibodies. However the expression mechanistic basis for the link between MSI genetic status and high efficiency of anti-PD-1 antibodies is not clear. Here, evidences from 7 expression datasets showing that MSI subtype colon cancer patients maintain immune activated statues, and PD-L1, IFN-γ, IFN-γ associated immune gene signature and transcription factor IRF1 are highly expressed in the MSI subtype colon cancer patients. While TGFβ and Wnt-β-catenin signaling pathways, which inhibit the sensitivity of immune therapies, are inactivated in MSI subtype colon cancer patients. Those results provide critical links between MMR deficiency and PD-1 checkpoint blockade therapeutic sensitivity in mRNA expression level.

Due to the direct functions of T cells as well as natural killer cells in eliminating tumor cells, the infiltration of those two cell types in tumor environment is well studied. T cells as well as natural killer cells could activate the production of IFN-γ. And IFN-γ directly up regulates PD-L1 expression in the PD-1 checkpoint blockade responding tumor microenvironment [13]. However, the roles of M1 macrophage cells in tumor microenvironment are rather neglected. We find high infiltration of M1 macrophage cells in MSI subtype colon cancer patients. And more likely, M1 macrophage cells are more important than CD8 T cells and γδ T cells in determining the immune blockade therapies, as presented by the results that M1 macrophages infiltration fraction is negatively associated with MLH1 expression, but positively associated with PD-L1 expression in colon cancer patients.

Although, most knowing biomarkers, like PD-L1, IFN-γ, IFN-γ associated immune gene signature, TGFβ/Wnt-β-catenin signaling, and CD8 T cells infiltration could be identified from the globe expression of MSI subtype colon cancer patients, some biomarkers may be not involved in MSI expression signature. Like transcription factor MYC [36] and chromatin regulator PBRM1 [37], which are reported to determine the sensitivity of PD-1 blockade therapy showed no difference between MSI and MSS colon cancer patients in mRNA expression level. One possible reason is that changes of MYC and PBRM1 expression are mainly in protein level. Another explanation is that MYC and PBRM1 represent independent different biomarkers contrast to MSI status. Further studies will reveal the relationship of MYC, PBRM1 expression and MSI status.

Overall, our results provide the first multiple datasets integrated description of globe expression of MSI subtype of colon cancer and its relationship with cancer immunotherapy. This new understanding of the molecular mechanisms underlying MSI subtype colon cancer patients should help the development of strategies to improve prognosis prediction and therapeutic efficacy.

## Conclusions

MSI subtype colon cancer patients are immune-hyper activated. Wnt-β-catenin and TGFβ signaling pathways are negatively related with MSI status. The immune-profiles in MSI subtype colon cancer are contributed by M1 macrophage infiltration in the tumor environment.

## Additional file

Additional file 1: **Figure S1.** Descriptions and validation of the datasets used in this study. (a) Table showed the detailed 7 GEO datasets used for MSI and MSS study in this paper. (b) Box plots showed the MLH1 expressions in 7 GEO datasets. (c) Table showed the detailed 6 GEO datasets used for cibersort fraction of 22 immune associated cell types studied in this paper. **Figure S2.** IFN-γ signature genes are not activated in MSI subtype colon cancer cell lines. (a) Heatmap demonstrated the regulated genes in MSI subtype colon cancer cell lines. (b) Box plots showed the PARP1 and PARP2 expressions in 7 GEO datasets. (supplementary data to Fig. 6d). **Figure S3.** MSI subtype colon cancer patients are with high M1 macrophage infiltration. (a) Un-supervised clustering heatmaps showed the cibersort fraction of 22 immune associated cell types in MSI and MSS colon cancer patients in GSE13294. (supplementary data to Fig. 7a). (b) Box plots showed the cibersort fraction of CD8 T cells in 4 GEO datasets. *P* values showed the difference of cibersort fraction of CD8 T cells between MSI and MSS patients determined by Student’s t test. (c) Box plots showed the cibersort fraction of γδ T cells in 4 GEO datasets. (PDF 2753 kb)

## Data Availability

All the data and softwares used in this paper are available as mentioned in Methods.

## Abbreviations

GZMA: granzyme A; PRF1, perforin
MMR: MisMatch Repair
MSI: Microsatellite instability
PD-1: Programmed cell death protein 1
PD-L1: PD-1 ligand
