# Supplementary material for: Analysis of the transcriptomic features of microsatellite instability subtype colon cancer
Source: BMC Cancer. 2019 Jun 20;19:605. doi: 10.1186/s12885-019-5802-2 (PMC6585086; doi:10.1186/s12885-019-5802-2)
Supplement: Supplementary file 1 — Figure S1. Descriptions and validation of the datasets used in this study. (a) Table showed the detailed 7 GEO datasets used for MSI and MSS study in this paper. (b) Box plots showed the MLH1 expressions in 7 GEO datasets. (c) Table showed the detailed 6 GEO datasets used for cibersort fraction of 22 immune associated cell types studied in this paper. Figure S2. IFN-γ signature genes are not activated in MSI subtype colon cancer cell lines. (a) Heatmap demonstrated the regulated genes in MSI subtype colon cancer cell lines. (b) Box plots showed the PARP1 and PARP2 expressions in 7 GEO datasets. (supplementary data to Fig. 6d). Figure S3. MSI subtype colon cancer patients are with high M1 macrophage infiltration. (a) Un-supervised clustering heatmaps showed the cibersort fraction of 22 immune associated cell types in MSI and MSS colon cancer patients in GSE13294. (supplementary data to Fig. 7a). (b) Box plots showed the cibersort fraction of CD8 T cells in 4 GEO datasets. P values showed the difference of cibersort fraction of CD8 T cells between MSI and MSS patients determined by Student’s t test. (c) Box plots showed the cibersort fraction of γδ T cells in 4 GEO datasets. (PDF 2753 kb) [file 12885_2019_5802_MOESM1_ESM.pdf]

Figure.S1

a

Datasets	MSI	MSS	Patform	PubMed_ID
GSE13067	N=11	N=63	GPL570 Affymetrix Human Genome U133 Plus 2.0 Array	PMID:19088021
GSE13294	N=78	N=77	GPL570 Affymetrix Human Genome U133 Plus 2.0 Array	PMID:19088021
GSE18088	N=19	N=34	GPL570 Affymetrix Human Genome U133 Plus 2.0 Array	PMID:21465190
GSE24551	N=21	N=110	GPL5175 Affymetrix Human Exon 1.0 ST Array	PMID:21619627
GSE26682	N=18	N=119	GPL96 Affymetrix Human Genome U133A Array	PMID:21300766
GSE39084	N=16	N=54	GPL570 Affymetrix Human Genome U133 Plus 2.0 Array	PMID:25083765
GSE41258	N=44	N=166	GPL96 Affymetrix Human Genome U133A Array	PMID:19359472

b

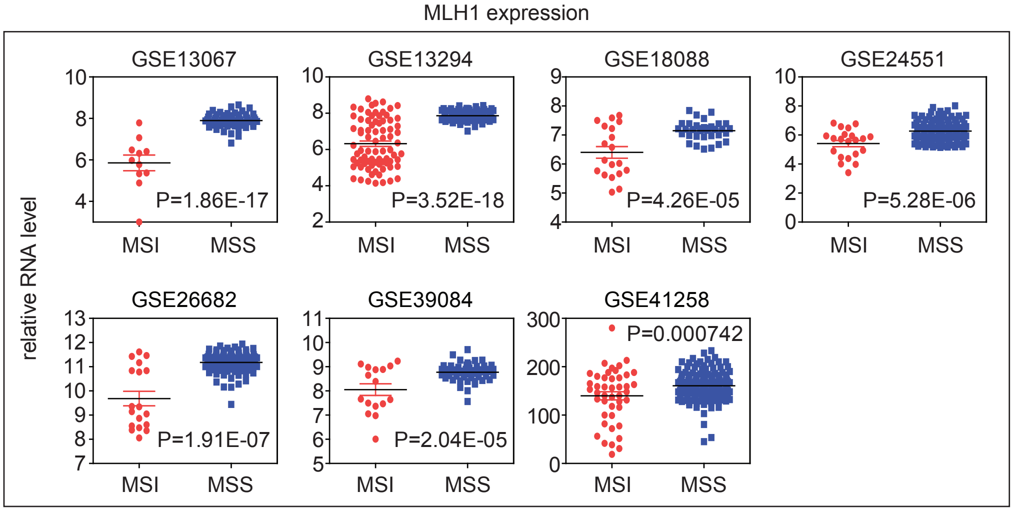

c

Datasets	Patient number	Platform
GSE14333	N=290	GPL570 Affymetrix Human Genome U133 Plus 2.0 Array
GSE17536	N=232	GPL570 Affymetrix Human Genome U133 Plus 2.0 Array
GSE33113	N=90	GPL570 Affymetrix Human Genome U133 Plus 2.0 Array
GSE39582	N=566	GPL570 Affymetrix Human Genome U133 Plus 2.0 Array
GSE24551	N=160	GPL5175 Affymetrix Human Exon 1.0 ST Array

Figure.S2

a

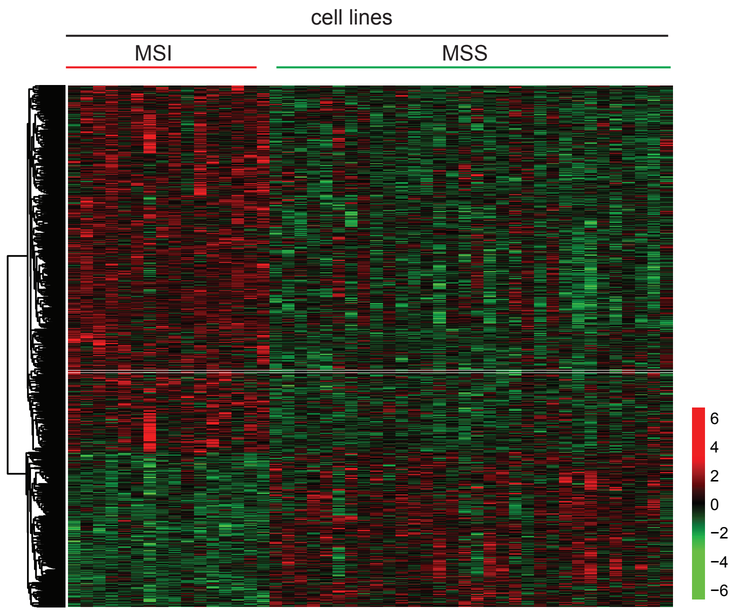
cell lines
MSI
MSS
6
4
2
0
−2
−4
−6

b

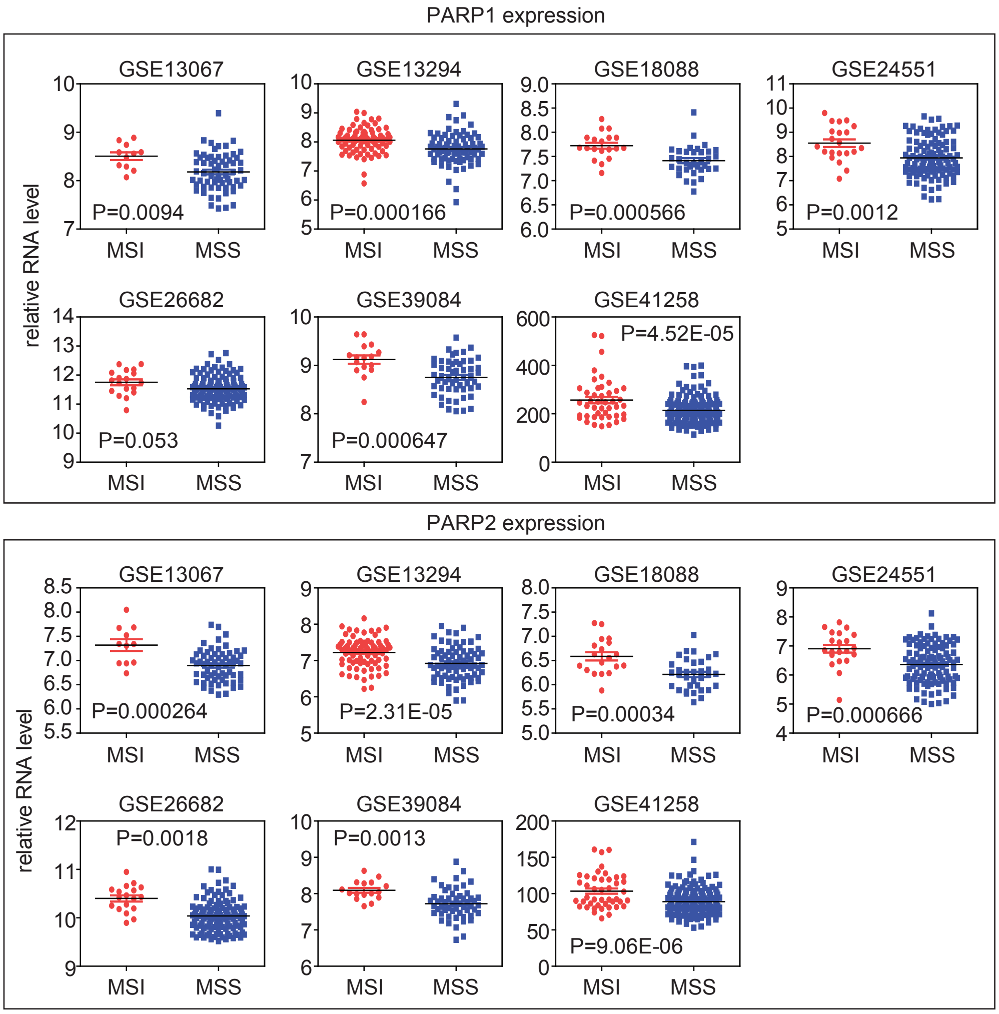
PARP1 expression
relative RNA level
GSE13067
P=0.0094
GSE13294
P=0.000166
GSE18088
P=0.000566
GSE24551
P=0.0012
GSE26682
P=0.053
GSE39084
P=0.000647
GSE41258
P=4.52E-05
MSI
MSS
PARP2 expression
relative RNA level
GSE13067
P=0.000264
GSE13294
P=2.31E-05
GSE18088
P=0.00034
GSE24551
P=0.000666
GSE26682
P=0.0018
GSE39084
P=0.0013
GSE41258
P=9.06E-06
MSI
MSS

Figure.S3

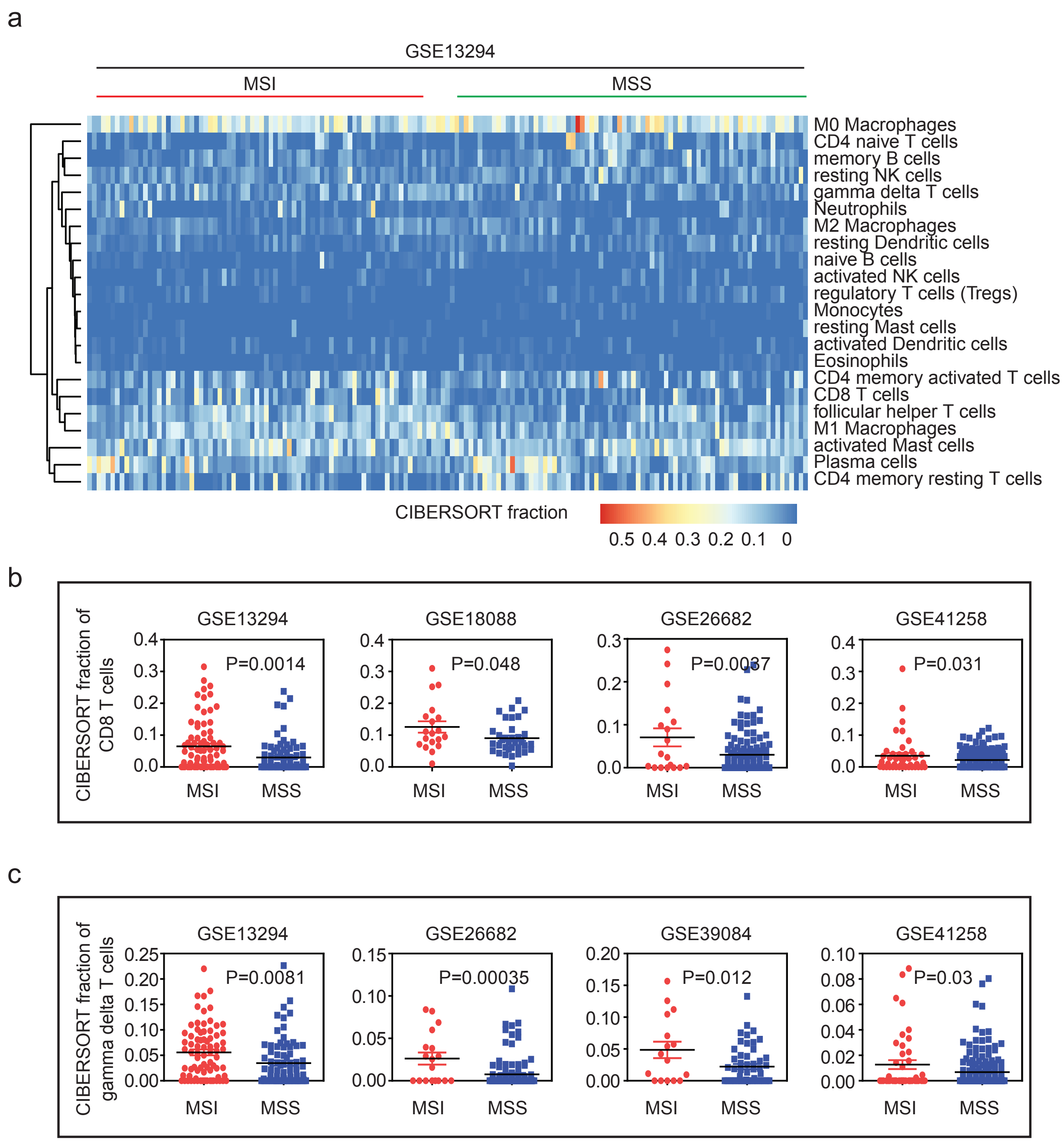
a
GSE13294
MSI
MSS
M0 Macrophages
CD4 naive T cells
memory B cells
resting NK cells
gamma delta T cells
Neutrophils
M2 Macrophages
resting Dendritic cells
naive B cells
activated NK cells
regulatory T cells (Tregs)
Monocytes
resting Mast cells
activated Dendritic cells
Eosinophils
CD4 memory activated T cells
CD8 T cells
follicular helper T cells
M1 Macrophages
activated Mast cells
Plasma cells
CD4 memory resting T cells
CIBERSORT fraction
0.5 0.4 0.3 0.2 0.1 0
b
CIBERSORT fraction of CD8 T cells
GSE13294
P=0.0014
GSE18088
P=0.048
GSE26682
P=0.0037
GSE41258
P=0.031
MSI
MSS
c
CIBERSORT fraction of gamma delta T cells
GSE13294
P=0.0081
GSE26682
P=0.00035
GSE39084
P=0.012
GSE41258
P=0.03
MSI
MSS
